# Spinal Accessory Nerve Injury following Spinal Adjustment: Case Report and Literature Review of the Outcome of Accessory Nerve Pathology as Result of Blunt Trauma (Spinal Accessory Nerve Palsy after Spinal Adjustment)

**DOI:** 10.1155/2024/7440745

**Published:** 2024-02-29

**Authors:** Sulaiman Alanazi, Areej M. Alawfi, Bander S. Alrashedan, Reem A. Almohaini, Majed M. Shogair, Talal A. Alshehri

**Affiliations:** ^1^Department of Orthopedic Surgery, Ministry of Health (MOH), King Saud Medical City, Ulaishah, Al Imam Abdul Aziz Ibn Muhammad Ibn Saud, 12746 Riyadh, Saudi Arabia; ^2^College of Medicine, Imam Mohammad Ibn Saud Islamic University, Othman Ibn Affan Street, 11432 Riyadh, Saudi Arabia

## Abstract

Spinal accessory nerve palsy (SANP) is rare and is commonly presented following iatrogenic injury. Their diagnosis is often missed on initial presentation. Injury following blunt trauma is rare, with few cases reported in literature describing blunt-associated SANP and their treatment and recovery. We present and discuss a case of SANP following an aggressive soft tissue adjustment by an uncertified individual that has been responsive to nonsurgical measures over 18 months. We also reviewed the related literature on similar cases that were presented as result of direct pressure on the nerve from soft tissue manipulation or heavy lifting and their outcome following treatment. Chiropractic is generally a safe complimentary medicine and must only be practiced by trained personnel. We found that blunt-caused SANP injuries should initially be treated conservatively as they are likely to respond and recover unlike when presented following invasive trauma accordingly to what we found in literature.

## 1. Introduction

Spinal accessory nerve supplies fibers of sternocleidomastoid and trapezius muscle which functions as a significant scapular stabilizer and contributes to scapulothoracic rhythm by elevating, rotating, and retracting the scapula [1, 2]. The incidence of spinal accessory nerve palsy (SANP) is uncommon with the most reported etiology being iatrogenic invasive trauma following surgical dissection around the neck. Other remaining etiologies reported were due to penetrating trauma and blunt trauma following direct impact following aggressive soft tissue manipulation, lifting heavy objects, and rarely due to idiopathic cause [3–7]. Patients with SANP may present with pain, loss of active shoulder abduction, winging of the scapula, and atrophy of the trapezius muscle causing asymmetry of the shoulder blades [8, 9]. Establishing the diagnosis of SANP can be a clinical challenge, and such diagnosis should include a detailed history and careful physical exam and magnetic resonance imaging (MRI). Additional nerve conduction testing can help diagnosis and follow-up the recovery [6]. Management of such pathology is known to be poorly responsive to treatment options which ranges from nonsurgical supportive measures by way of physical therapy or surgically by way of nerve exploration, tendon transfer, or scapulothoracic fusion [3–9].

In this paper, we present and discuss a case of blunt-caused SANP following an aggressive soft tissue manipulation by an uncertified practitioner that has been responsive to nonsurgical treatment and review literature of similar cases that were presented because of direct compression on the nerve from soft tissue manipulation or heavy lifting.

## 2. Case Presentation

**Figure 1:**
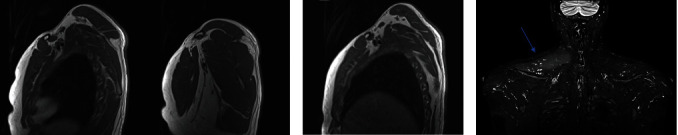
(a) Sagittal MRI cut showing clear atrophy of the trapezius muscle compared to the (b) left side. (c) Coronal fat suppression MRI showing edema affecting right trapezius muscle.

**Figure 2:**
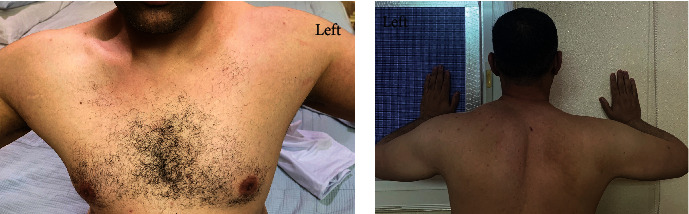
Clinical images of the patient 4 months from the trauma showing right-sided trapezius atrophy with prominent ipsilateral clavicle from the (a) front and lateral scapular winging and asymmetry due to flattening of the periscapular muscle region compared to the contralateral side on (b) wall-push test.

**Figure 3:**
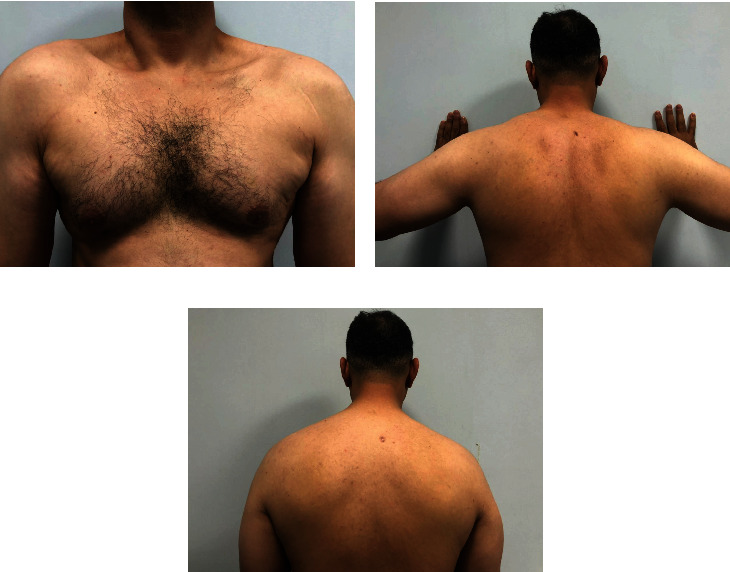
Clinical images of the patient at 1 year from the trauma showing improvement of the right-sided trapezius atrophy with residual prominence of the ipsilateral clavicle from the (a) front and improvement of muscle wasting of the right periscapular muscles compared to clinical photographs taken on presentation while (b) doing wall-push test and while (c) standing still.

A 43-year-old male recreational bodybuilder who is not known to have any medical illnesses presented with a complain of muscle soreness between the shoulder blades. Two months prior to presentation, he underwent spinal adjustment/deep tissue release by his personal trainer who is uncertified at doing this type of procedure. Manipulation was carried out by pressing between the patient's shoulder blades using his feet while the patient is grounded with both shoulders abducted 90 degrees from his body which led to severe chest pain and shortness of breath. He was treated conservatively at that time and later presented to us with worsening weakness and a notable change in the appearance of his back with associated shoulder pain. MRI was requested and showed tendinopathy of supraspinatus and subscapularis tendons that were thought to be the cause of his symptoms and were managed with physical therapy and analgesics. During rehabilitation, shoulder pain improved; however, the patient noticed worsening of his back appearance and drooping of the right shoulder blade with muscle wasting of the supraclavicular region over a 2-month period. A concern regarding SANP was raised, and an MRI was requested which showed inflammatory changes and wasting of the right trapezius compared to the left side making our diagnosis certain (Figure 1). He was found to have classic signs of SANP including muscle wasting and lateral winging of the scapula with a restricted active movement that was improved with scapular stabilization (Figure 2). The patient was started on physiotherapy and the use of transcutaneous electrical nerve stimulation (TENS). Electromyography/nerve conduction studies (EMG/NCS) were performed 1 year after the injury and showed weak spontaneous activity of right trapezius and mild reinnervation amplitude of right accessory nerve that is 50% of the left side. Clinically, the patient reported notable self-rated improvement and has resumed weightlifting activity but not to the same level he used to prior to the injury (Figure 3). He has been followed for 18 months now and has shown excellent self-reported recovery and good muscle power with minimal functional limitation.

## 3. Discussion

A clear relationship between SANP and a history of neck dissection or lymph node excision was reported in the literature; in addition, other causes have been reported due to blunt trauma, such as deep tissue massage, lifting heavy objects, motor vehicle accidents, falls, and, more rarely, spontaneous onset [3–10]. In our case, the patient underwent spinal adjustment by a self-claimed experienced chiropractic specialist. Chiropractic is a form of alternative medicine that involves manual spinal and joint manipulation to alleviate pain and improve function that has recently gained popularity worldwide [11]. It is generally considered safe when carried out by a certified practitioner with few reports on complications caused by this type of treatment [11–14]. It is under complementary medicine and alternative medicine category and follows rules and regulations in the country of Saudi Arabia by the National Center for Complementary and Alternative Medicine [15]. Nerve injuries are believed to be due to certain maneuvers or excessive force during spinal adjustments that may lead to nerve compression, stretching, or traction. Notably, from the available literature, these cases are relatively rare.

A case series concluded signs and symptoms of SANP are droopy shoulder, trapezius wasting, scapular dyskinesis, and loss of the affected shoulder abduction [3]. In this case, the patient started to have the classical signs of SANP over time as the diagnosis was not made on initial presentation. The difficulty was due the MRI finding of rotator cuff tendinopathy linked to weight lifting and being a military recruit. The patient was informed that spontaneous recovery is uncommon according to the current literature; however, the pathology tends to present following invasive trauma unlike in his presentation. In the available literature discussing similar cases in terms of the nature of the causative pathology being high compressive pressure leading to nerve palsy, we found that they were all managed nonsurgically and showed some improvement; however, in the majority, residual weakness or functional limitation was evident on last follow-up (Table 1) [4, 5, 7, 10].

Coulter J et el. reported a case with SANP following tight pressure from a worn robe used for lifting climbing gear that was treated conservatively and showed near full recovery at 6 months [4]. Aksoy I et al. reported a similar case to ours in terms of the exact mechanism of injury which was followed for 2 years and showed residual strength and functional deficit. They conducted electrodiagnostic testing which showed fibrillation potentials in EMG [7]. Yoon JR et al. reported a case caused by deep soft tissue massage; her EMG/NCS showed significant changes. Their case recovered in 2 months following conservative treatment [10].

From the available literature, outcomes following SANP as result of compressive injury are not clear and are somewhat unpredictable when it comes to recovery with all cases reviewed along with ours showing notable response to conservative measures. We believe that the main reason behind that is the different forces causing the trauma and the regeneration potentials between a patient and another is variable.

## 4. Conclusion

Spinal adjustment or soft tissue manipulation must only be carried by experienced personnel as they might lead to devastating nerve pathology. Workup of a patient with shoulder pain should be detailed as signs of SANP might be masked by other incidental findings and progressive nature of the pathology. Outcomes following SANP are variable, and conservative management should be the first line of treatment for blunt-caused SANP as they can recover over time.

## Figures and Tables

**Table 1:** SANP cases reported in literature that were attributed to compressive trauma.

Case	Date	Age Gender	Side	NCS/EMG	Mechanism of injury	Management	Course	Duration
Case 1 Braybrooke J et al. [5]	2003	74 Male	Right	Delay in motor potentials	Lifting a heavy object	N/A	N/A	N/A
Case 2 Aksoy I et al. [7]	2009	38 Female	Left	Fibrillation and reduced motor units' potentials	Deep tissue massage	Nonsurgical management	Complete resolution of pain Residual strength and function deficit	2 years
Case 3 Coulter J et al. [4]	2015	31 Male	Right	N/A	Wearing tight robe	Nonsurgical management	Complete resolution of pain and strength and deformity	6 months
Case 11 Yoon JR et al. [10]	2018	42 Female	Right	Delayed latency and decreased amplitude	Deep tissue massage	Nonsurgical management, neuromuscular electrical stimulation	Complete recovery of pain and strength Residual deformity was not documented	2 months

## Data Availability

Any required links or identifiers for the data are present in the manuscript as described without any edits that could potentially affect the quality of the scientific message provided.
